# Implementation and quality assessment of a clinical orthopaedic registry in a public hospital department

**DOI:** 10.1186/s12913-020-05203-8

**Published:** 2020-05-09

**Authors:** Binglong Lee, Milad Ebrahimi, Nalan Ektas, Chee Han Ting, MacDougal Cowley, Corey Scholes, Christopher Bell

**Affiliations:** 1 Orthopaedics Department, QEII Jubilee Hospital, Coopers Plains, Queensland Australia; 2 EBM Analytics, Crows Nest, NSW 2065 Australia

**Keywords:** Registry, Knee, Shoulder, Validity, Consistency, Compliance, Data quality

## Abstract

**Background:**

The aim of this study was to demonstrate a novel method of assessing data quality for an orthopaedic registry and its effects on data quality metrics.

**Methods:**

A quality controlled clinical patient registry was implemented, comprising six observational cohorts of shoulder and knee pathologies. Data collection procedures were co-developed with clinicians and administrative staff in accordance with the relevant dataset and organised into the registry database software. Quality metrics included completeness, consistency and validity. Data were extracted at scheduled intervals (3 months) and quality metrics reported to stakeholders of the registry.

**Results:**

The first patient was enrolled in July 2017 and the data extracted for analysis over 4 quarters, with the last audit in August 2018 (*N* = 189). Auditing revealed registry completeness was 100% after registry deficiencies were addressed. However, cohort completeness was less accurate, ranging from 12 to 13% for height & weight to 90–100% for operative variables such as operating surgeon, consulting surgeon and hospital. Consistency and internal validation improved to 100% after issues in registry processes were rectified.

**Conclusions:**

A novel method to assess data quality in a clinical orthopaedic registry identified process shortfalls and improved data quality over time. Real-time communication, a comprehensive data framework and an integrated feedback loop were necessary to ensure adequate quality assurance. This model can be replicated in other registries and serve as a useful quality control tool to improve registry quality and ensure applicability of the data to aid clinical decisions, especially in newly implemented registries.

**Trial registration:**

ACTRN12617001161314; registration date 8/08/2017. Retrospectively registered.

## Background

Clinical registries serve as repositories for the collection of patient, treatment and outcomes data, and are valuable tools for determining the natural history of a disease or condition, evaluating the clinical performance and cost-effectiveness of healthcare services, and monitoring the safety and quality of patient care [1]. While national orthopaedic registries have been implemented in numerous countries to capture survival data for prostheses, they are limited with respect to capture of potentially modifiable risk factors for revision, and typically do not capture clinically relevant outcomes such as complications and patient-reported outcome measures (PROMs) [2]. Local registries are in a key position to provide greater insight into the clinical performance of individual surgeons, hospital departments or local health districts by capturing a greater variety of data on a more representative patient population, and thus provide more relevant clinical information pertaining to specific outcomes of interest [3]. However, local, multi-cohort registries are not commonplace, particularly within the domains of sports injuries and treatments, including shoulder and knee reconstructive surgeries.

Implementation of a registry is a complex and resource intensive task, and requires methodical planning, execution and management, with a clear pre-defined purpose and dataset [3]. The validity of a registry analysis is highly-dependent on the quality of the dataset, and requires a framework for appropriate data collection and data quality assurance [4], to limit bias in patient selection, information collected, or confounding [5, 6] and minimise inaccurate and incomplete data [4]. Quality assessment of registry data has been traditionally reported for established national and hospital arthroplasty registries via regular auditing of two quality domains, data completeness [7–12] and data accuracy [8, 9, 11, 12], with data completeness generally defined as the proportion of necessary data to be captured that has actually been registered in the registry, while data accuracy referred to as the extent to which that data is representative of the truth [4]. More recently, a newer model of validation was proposed, with assessment of quality across three domains - adherence, completeness, and accuracy, for an institutional arthroplasty registry [13]. However, the definitions of quality domains and methods for assessment of quality metrics are often ambiguous or unclear, making comparisons across studies difficult, and there remains a lack of consistent information to establish a reasonable framework and benchmark for assessing quality metrics in the implementation of a local hospital orthopaedic registry with multiple defined cohorts.

The aims of this study were therefore threefold: Firstly, to report on the implementation of a quality controlled multiple-cohort clinical orthopaedic registry at a single public hospital, secondly to describe a novel model of registry quality assessment for a multiple-cohort registry, and thirdly to report the changes in quality metrics of the registry during its initial operation. We hypothesise that the framework for data collection and established quality system would detect issues and contribute to quantifiable improvements in registry quality over time.

## Methods

### Registry implementation

A clinical research registry was planned and implemented within a public hospital department, for the collection of clinical data and outcomes of patients presenting to the senior author and undergoing surgical treatment for shoulder and knee pathology. Ethical approval for the registry was granted by the Metro South Health Human Research Ethics Committee (HREC/16/QPAH/732), and the study was registered on the Australian New Zealand Clinical Trials Register, ACTRN12617001161314). A framework for the registry was established at its onset and comprised six observational, prospective cohorts consisting of shoulder and knee pathologies that were of research interest to the senior author. Each cohort was defined by a pathology and primary diagnosis appropriate for surgery (**Supplementary file** **1**), as well as a research and analysis plan.

A core dataset, comprising a minimum list of variables to be collected [4], was composed for each registry cohort (**Supplementary file** **2**). The core dataset consisted of common variables pertaining to patient demographics, diagnosis and surgical details, clinical evaluations observed prior to, during surgery and at follow up, as well as cohort specific questionnaires to capture region, pathology and treatment related patient reported outcomes measures (PROMs). The data characteristics for each variable, including data source, timepoints for data collection, as well as allocation of responsibility for the collection and entry to registry database, were defined for the core dataset of each registry cohort.

Data collection methods were developed with clinical administrative staff and clinicians to ensure transparency of processes, and were piloted prior to implementation. The data collection protocol was documented in a registry manual for reference and training purposes, and stored on a secure website accessible by key staff contributing to the registry. A quality assurance plan comprising a quality framework and auditing schedule (described further in the following sections) was formulated to ensure data captured to the registry was of acceptable research quality. Iterative changes to registry processes were captured via updates to the registry manual.

### Patient Recruitment

The primary recruitment pathway for enrolment of patients to the registry was via consultation with the senior author during outpatient clinics. An initial diagnosis was formed on patients presenting with shoulder or knee pathologies as per the standard clinical pathway. Patients were screened into the appropriate cohort based on primary diagnosis (**Supplementary file** **1**) and indication for surgery. Patients were recruited using an opt-in strategy, and provided written informed consent for the collection of clinical data for research purposes. General exclusion criteria were a patient’s unwillingness to participate in data collection or revocation of consent for research use of personal data.

### Data collection protocol

The data collection team comprised clinical administrative staff, clinicians and the registry custodians, who were responsible for ongoing management of the clinical registry. Communication between the data collection team was established using live electronic messaging.

**Fig. 1:**
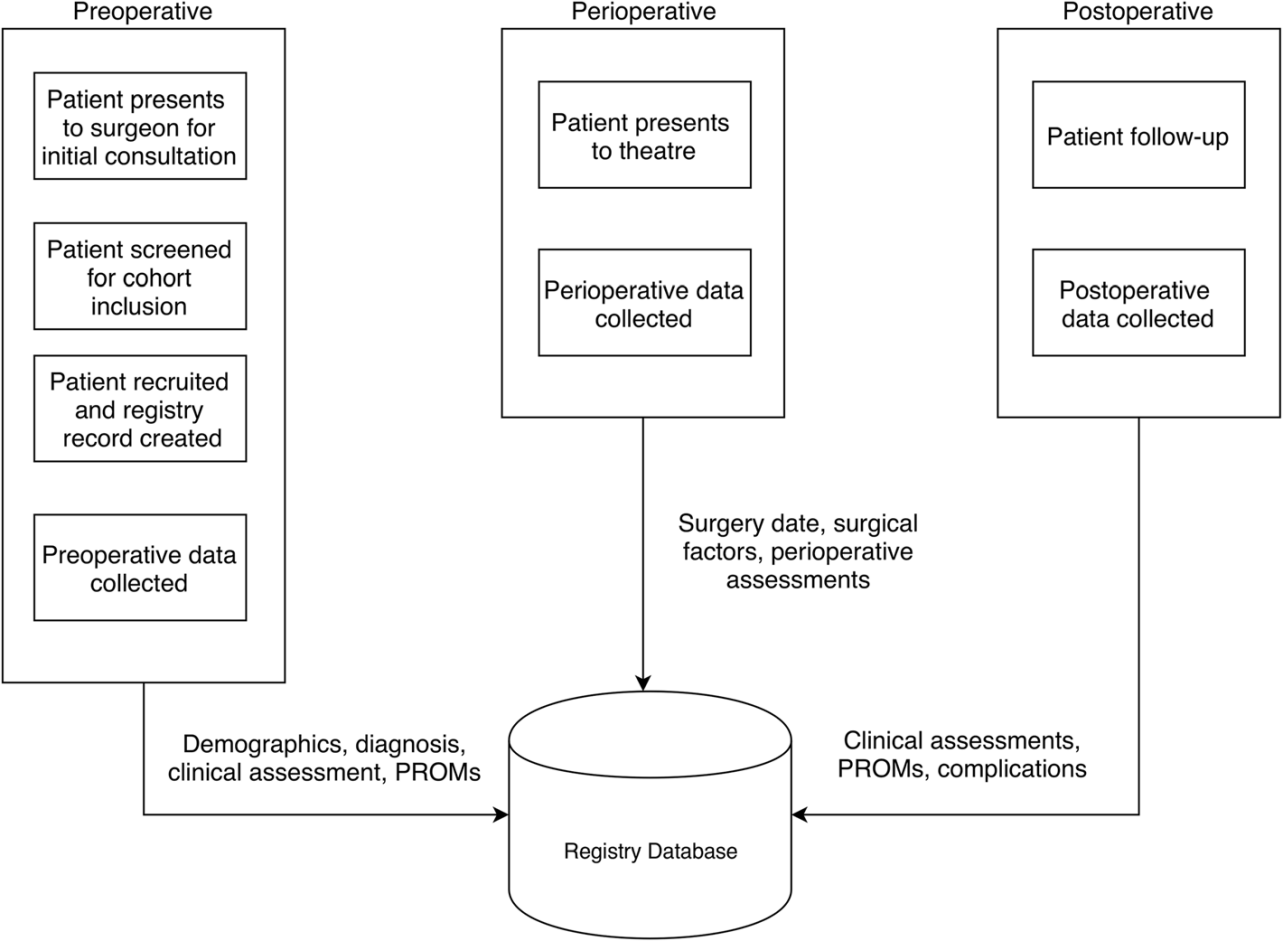
Data collection process outlining patient screening, recruitment and collection of cohort data

The data collection protocol (Fig. 1) included collection of preoperative, perioperative and postoperative data as per the individual cohort-specific core dataset. A treatment record for a patient was created by the registry custodians within the registry’s database software (Socrates v3.5, Ortholink Pty Ltd., Aus) upon confirmation of diagnosis, cohort and registry recruitment.

Data collection pre- and postoperatively involved the completion of standardised patient questionnaires that were collected by the clinical team and scanned electronically to the registry custodians. Data from the scanned forms were manually entered into the registry’s software under the patient’s treatment record and stored on the electronic database. Surgical findings and procedure details were entered directly into the software on the day of the procedure.

Postoperatively, registry participants returned to the outpatient clinic for scheduled follow up. Weekly outpatient appointment lists were cross checked against treatment records by the registry custodians to identify patients who were due for data collection. These patients were flagged to the surgeon’s team for collection of clinical data and questionnaires specific to the respective cohort and postoperative time point.

### Model of quality assessment

The quality assurance framework consisted of three quality assessment domains, and definitions of auditing schedule, roles and reporting lines to assess the accuracy and quality of the data recorded in the registry. Data were extracted at monthly to quarterly intervals by the registry custodians during the implementation of the registry as per the schedule on the quality assurance framework (Table 1). Quality metrics were reported to stakeholders within the Registry Governance Steering Committee, which included participating surgeons and primary investigators, the registry custodian team and representatives from clinical staff or information technology personnel as needed, to identify problem areas, refine collection and organisation procedures, as well as address any gaps in datasets that could be retrieved retrospectively from clinical records.

**Table 1:** Quality Domain assessments

Quality domain	Completeness		Consistency	Validity	
Level of assessment	Registry	Cohort	Registry	Internal	External
**Domain objective**	Assess the capture of participants to the registry	Assess the capture of data within specified cohorts	Assess the accuracy of placement of patients into correct cohorts; identify issues with data capture and entry (e.g. transcription errors)	Assess the accuracy of patient-specific data records as a true reflection of individual clinical data and reported outcomes	Assess the reliability of aggregated cohort data against benchmarks determined from evidence based literature
**Method of assessment**	Ratio of treatment records in the registry to number of patients eligible for participation in the registry. Calculated by checking archived consult lists containing patients assigned to a cohort against treatment records stored in the electronic database.	Ratio of data captured for patients’ treatment records compared to the total number of variables within the CDS for each cohort. Calculated by dividing the number of patients at time (x) with data (i) available, by the number of patients eligible for collection of (x)(i). Assessed for all treatment records entered into the registry.	All treatment records were retrieved and diagnosis was checked against cohort inclusion and exclusion criteria. Any cohort assignment that did not match the diagnosis was flagged and the contributing surgeon notified. Outlier analysis utilising quartiles method was performed on current age, age at surgery, height and weight. Assessed for all treatment records that had a diagnosis entered into the registry.	Validate individual patient data records to original data / patient submitted forms. Determined by comparing source data and data transcribed to the registry software. Data validation performed by a registry custodian member who was independant to data entry. Assessed for all treatment records entered into the registry.	The highest quality evidence of appropriate patient outcomes were used to benchmark aggregated cohort PROMs data. Assessed for all treatment records with PROMS data captured to the registry.
**Audits performed during pilot period (July 2017 - Aug 2018)**	12	6	6	4	4
**Benchmark**	90% [9, 13]	90% [9, 13]	95% set internally by registry custodian team	90% [9, 13]	Varied depending on PROM

### Quality domains

A model of quality assessment was implemented to report quality metrics for all records across the following domains: completeness, consistency and validity. Definitions of domain objectives, methods of assessment and auditing schedules are presented in Table 1. For the completeness domain, quality assessment was conducted over two levels - at the registry-level to report quality measures for the registry as a whole, and at the cohort-level, to provide a more comprehensive quality control strategy. Validity of the registry was assessed internally against original data sources and externally against benchmarks determined from evidence-based literature.

## Results

A total of 189 patients were included for analysis and their demographics are shown in Table 2. The first patient was enrolled in July 2017 and the data extracted for analysis after the last audit completed in August 2018. New patients were enrolled at presentation throughout the study period, with 33 recruited in quarter 1, 62 in quarter 2, 60 in quarter 3, and 34 in quarter 4. Data collection spanned clinical, surgical, functional and imaging data, as well as postoperative complications and PROMs.

**Table 2:** Summary of patient demographics captured in the registry during the pilot period (*N* = 189). IQR - Interquartile range; BMI – Body mass index

Male (%)	63
**Presentation (%)**	
**Knee**	41
**Shoulder**	59
**Age (median, years)**	29 (IQR = 23–46)
**Height (median, m)**	1.74 (IQR = 1.7–1.8)
**Weight (median, kg)**	81 (IQR =72–99.3)
**BMI (median, kg/m**^**2**^**)**	27.2 (IQR = 24.6–31.1)

Quality auditing of registry completeness against source lists from the hospital revealed an overall capture rate of 96.8 and 94.3% treatment records in the first and second quarters, respectively. Discrepancies in registry completeness were detected by an internal validation audit revealing a lack of registry record for these patients, despite complete records for PROMs retrieved. Once these missing patients were accounted for, a capture rate of 100% was achieved in the third and fourth quarters.

**Fig. 2:**
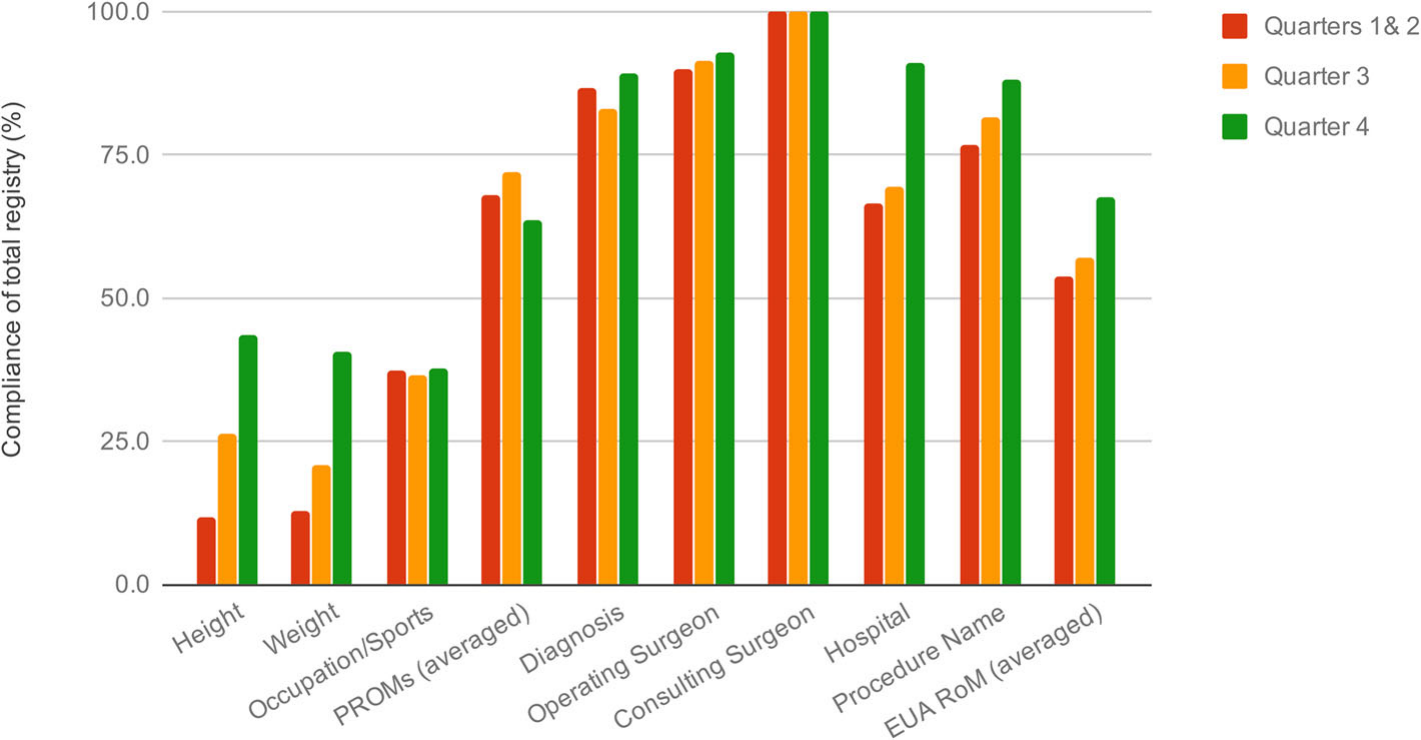
Preoperative completeness of registry variables common to all cohorts over the pilot period. EUA examination under anaesthesia, RoM range of motion

Individual patient cohort completeness for the predefined data sets was less accurate, ranging from 10 to 100% for common patient information across the registry such as height, weight and occupation/sport status and PROMs (Fig. 2). There was an upward trend in the rates quarter by quarter, with only a reduction in Quarter 4 for the PROMs.

**Fig. 3:**
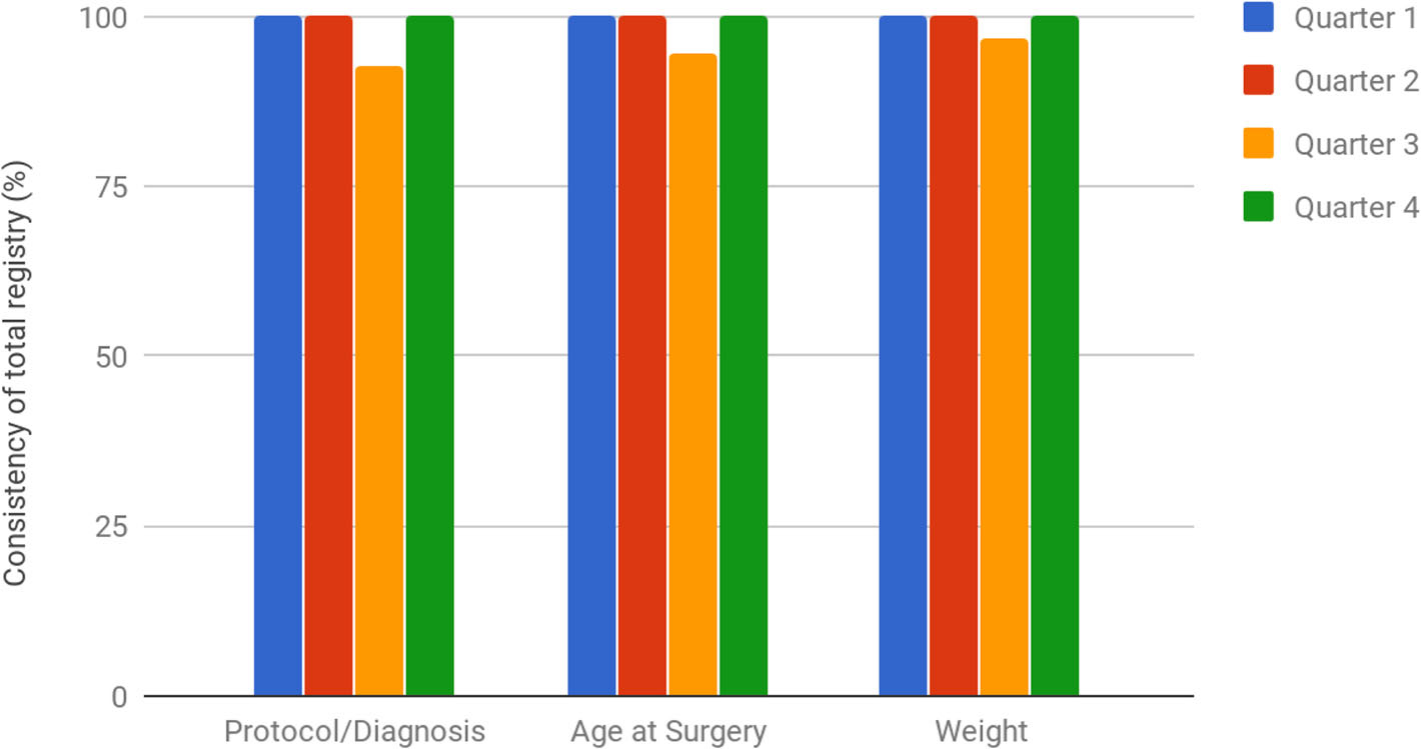
Consistency of data for variables common to all cohorts within the registry over the pilot period

Registry consistency on the other hand was at 100% in quarters 1, 2 and 4. There was a reduction in consistency in Quarter 3 (Fig. 3). Height and current age were also assessed and remained at 100% throughout.

**Fig. 4:**
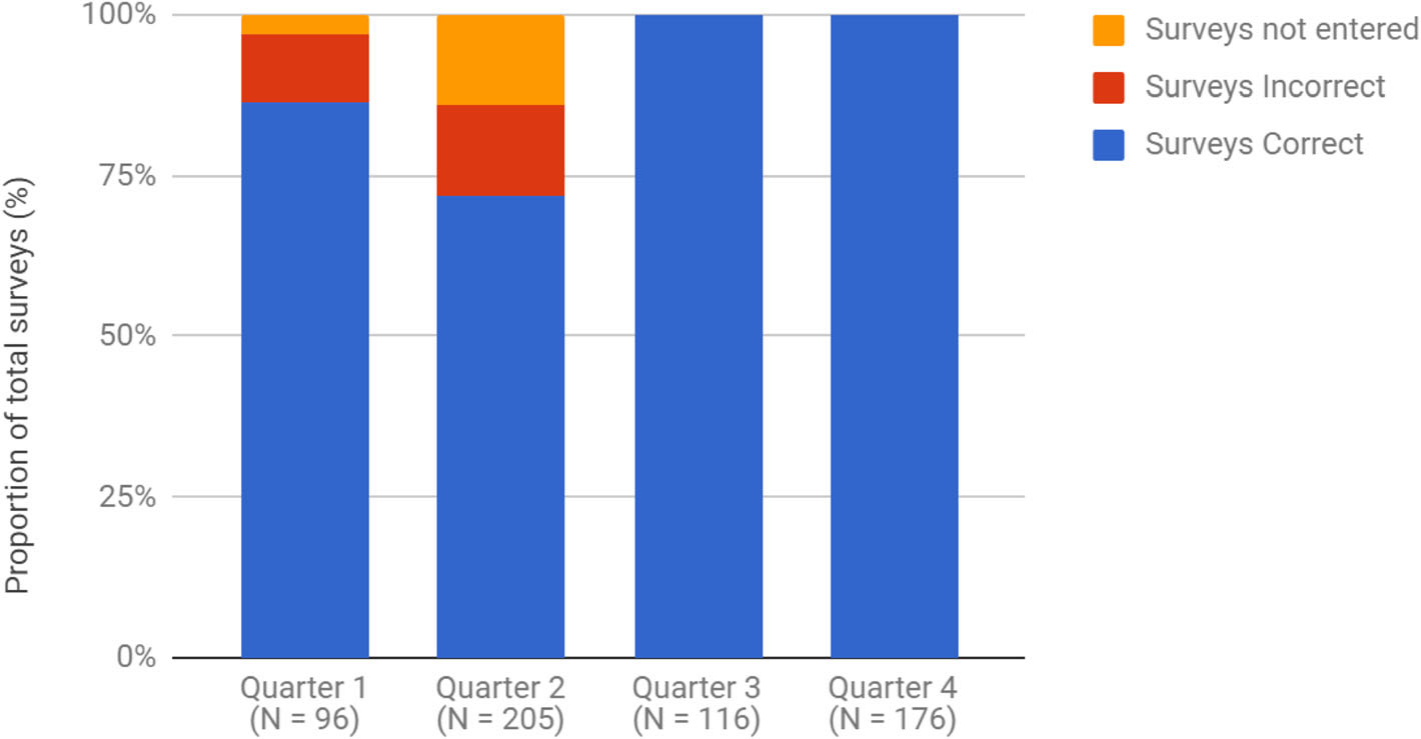
Internal validity of PROMs over the pilot period

Validation of digital registry records against source data was completed for patients returning paper forms (100% return rate) for PROMs (Fig. 4). Through Quarters 1 and 2, 3.1 and 14.1% of returned paper forms were not entered into the registry. Additionally, comparison between paper and software records indicated that by Quarter 1, 10.4% of surveys were transcribed incorrectly into the digital record, which increased to 14.1% by Quarter 2.

### Procedure evolution

A team approach was used to implement changes to the patient and clinical data capture processes. The first concerned the uploading of patient surveys to the registry. While patient questionnaires were initially scanned into the registry, the audit analysis indicated inadequate print quality, insufficient scan resolution and failure to follow the correct response format by patients were likely contributors to poor data quality. The process was altered from Quarter 3, whereby clinical staff scanned patient forms to a mutually accessible folder, with the research custodian team transcribing the data to the registry software.

The second change was in response to survey packs missing data variables (such as height and weight) and a reduction in PROMs data quality by Quarter 4. This was rectified by directly scanning the clinic pre-admission screening form, which contained these fields, for subsequent patients from Quarter 3. Data entry processes were also changed for Quarter 4, with restrictions placed on transcription of ambiguous responses and formalisation of the definitions of ambiguous patient responses. This was particularly problematic for PROMs containing visual analogue scales or questions with tables of responses. Both clinicians and patients were given updated instructions and additional education with regards to the PROMs forms. The instructions and layout of the forms were also modified to guide patients in completing the surveys more accurately.

Thirdly, mismatches were observed for some patients between the *cohort* they were placed within the registry and their recorded *diagnosis* in Quarter 3. Analysis revealed that the addition of new diagnoses to the registry software had not been updated simultaneously in the quality audit framework. In addition, outlier analysis revealed discrepancies in *age at surgery* and *weight* consistency in Quarter 3, which were caused by a defect within the registry software, which was subsequently addressed with the vendor. Communication between the onsite clinical team and the offsite registry custodian team evolved throughout the pilot period of the registry. A combination of file transfers to mutually accessible online folders, transfer of lists from hospital records and real-time communication through online instant messaging were phased in during the pilot period. The registry custodian team delivered daily lists of existing registry patients requiring surveys to be filled on the day of surgery or outpatient appointments to the clinical team as required. Messaging also allowed for real time alerts from clinicians regarding new patients to be added to the registry.

## Discussion

Orthopaedic registries are an important and effective tool for both research and improving patient care [1]. However, the applicability of registry data is dependent on its quality [4]. The implementation of the registry with a clear purpose is also crucial to ensure that the data collected can be fully utilised [3]. Bautista et al. [13] described a model of validation for a local arthroplasty registry via their definition of “adherence”, “completeness” and “accuracy”. Our model of quality assessment is a novel three-pronged approach to evaluate the completeness, consistency and validity of patient data captured within a clinical registry. In essence, our completeness assessment covered both the “adherence” and “completeness” assessments proposed by Bautista and colleagues [13], while our appraisal of “accuracy” via a combination of consistency and validity auditing could be considered a more robust method. This novel approach, specifically with the assessment of registry consistency and external validity, has not been reported in contemporary literature as outlined in Table 3. We believe that these two additional auditing methods, not previously considered in the literature, lead to a more comprehensive assessment of registry data quality. Consistency of the data contained in the registry provided timely indications on the accuracy of data transfer or problems with data entry that may render the data unusable for analysis. External validation provides verification that PROM scores are being administered as intended, and determines whether the aggregated outcome of a treatment reflects broadly across all patients. However, comparison of present quality metrics to previously established registries requires careful consideration due to inconsistencies and disparity in the definition of terms referring to the completeness, adherence and accuracy of data (Table 3).

**Table 3:** Novelty of auditing methods introduced relative to contemporary literature. *CDS - core dataset*

Quality Audit	Completeness		Consistency	Validity	
Level of Assessment	Registry	Cohort	Registry	Internal	External
**Definition as per current study**	Ratio of treatment records in the registry to number of patients eligible for participation in the registry	Proportion of data captured for patients’ treatment records compared to the total number of variables within the CDS for each cohort	Accuracy of placement of patients into correct cohorts	Accuracy of data in registry validated against original data / patient submitted forms	Reliability of data against evidence based literature benchmarks
**Current study**	✔	✔	✔	✔	✔
Bautista et al. 2017 [13]	✔ “adherence”	✔ “completeness”		✔ “accuracy”	
Torre et al. 2017 [7]	✔ “Completeness” or “quality rate”				
Seagrave et al. 2014 [8]	✔ “registry completeness”	✔ “Cohort completeness” (Demographic, administrative, medical history, procedure and acute care details only. PROMs were not audited.)		✔ (“accuracy”)	
Barr et al. 2012 [9]	✔ “completeness”			✔ “accuracy”	
Espehaug et al. 2006 [10]	✔ “Completeness”				
Arthursson et al. 2005 [12]	✔ “Completeness”			✔ loosely described	
Fender et al. 2000 [11]	✔ “Completeness”			✔ “inaccuracies”	

Despite the lack of gold standard for completeness, consistency and validity in orthopaedic registries, rates above 90% [9, 13] or 95% [8] have been described as acceptable in the literature. This study reports a 100% capture rate at one year with respect to registry completeness, consistency and internal validation after deficiencies in data capture processes were addressed. Reports on registry completeness, in relation to the capture of eligible patients for participation in a clinical registry, are varied in the literature, ranging from 50 to 98.7% [7–9, 13]. Registry completion was determined to be highly dependent on the participation of both patients and staff to the collection of clinical data for monitoring purposes.

Capture of data for individual cohorts in the current registry implementation pilot was less accurate, ranging from 10 to 100%, with lower rates of capture primarily for the preoperative variables of height, weight and work status/activity level. As aforementioned in our results, the variables of height and weight had a particularly low completion rate in the first half of the pilot for two reasons; firstly, they were missing from the survey packs during the first quarter, and secondly due to inadequacies in the automated transcription function of the research software. This was rectified and led to some improvements as seen above, although completion rates remained low for the duration of the pilot. Survey of staff at the end of the pilot indicated some had not been made aware that they were responsible for collecting height and weight - this has since been rectified with changes made to the induction process. The work status/activity on the other hand was frequently left blank by patients. This may be due to the fact that an open-ended response was required. A change to a multi-choice checkbox would likely lead to a higher completion rate in the future [14]. In contrast, the Arthroplasty Clinical Outcomes Registry NSW (ACORN) reports 99.0–99.3% completeness of their single-cohort registry dataset [8]; however the improvements in cohort completeness trends are encouraging. The modifications to data capture processes have improved patient data as demonstrated in the later quartiles, in comparison to accuracy rates of 85.8 to 96.1% reported by other clinical registries [8, 13].

The present findings demonstrate the importance of detailed and regular auditing and reporting for data quality. The novel quality assessment methods proposed within this study enabled identification of causative issues such as problematic data entries, transcription errors and ambiguous patient responses to questionnaires, and facilitated the implementation of strategies to improve data collection processes, with demonstrable improvements in data quality. Ongoing audits also provided a feedback mechanism to assess the effectiveness of changes to registry data entry processes, leading to improvements in internal validation and registry completeness. Furthermore, non-quantifiable changes such as improving communication, education and training led to improvements in data quality as reflected by the high registry completeness rate observed, confirming communication as a key factor in the success of a quality controlled registry [9].

The present study has contributed important information to the planning and implementation of a comprehensive patient registry within a hospital department. However, the results should be interpreted in the light of its limitations. Firstly, our study was conducted over a relatively short period of about 12 months. This limited the improvement seen over the pilot period, especially with respect to cohort completeness, despite improvements made to the registry framework and its processes. A longer period of study could identify additional errors and allow more substantial improvements in data quality to be observed. Additionally, manual quality assessment on all records is not feasible for a larger cohort, so the approach listed above would need to be adapted once a newly implemented registry has been operating for some time. Further work is underway to report the quality of individual cohorts to provide insights into cohort-specific datasets and peculiarities associated with them will improve and maintain the quality of the registry.

In the future, the registry will transition to the use of electronic surveys which should assist with automating quality assessment and subsequently improve data quality. There is an emerging body of literature indicating the strength of mixed-mode capture of PROMs, with greater reliance on electronic methods [15–17]. With the advent of digital hospitals, we may also see data populated in accessible systems as a by-product of normal clinical activity. Additionally, a research nurse may also be of benefit serving as permanent personnel responsible for coordination of the registry. Ensuring PROMs surveys are completed accurately prior to a patient’s departure from the clinic would also have a large impact on cohort completeness. With time and refinement, more surgeons and other cohorts will be added to expand the registry within the department.

## Conclusions

The quality of data from a clinical registry underpins its impact in improving care standards. We have demonstrated the implementation of a quality controlled clinical orthopaedic registry within a public hospital system at its initial operation. A unique framework targeting multiple aspects of data completeness, consistency and validity paired with comprehensive, regular auditing and feedback contributed to superior data quality in a short time period. Improvements in registry quality over time can be clearly observed. This model can be replicated in other registries to improve clinical impact and ensure applicability of the data to aid clinical decisions, especially in newly implemented registries.

## Supplementary information

**Additional file 1: Supplementary file 1**: Registry cohorts and inclusion/exclusion criteria. Defines cohorts for pilot registry and contains detailed list of inclusion and exclusion criteria for each cohort.

**Additional file 2: Supplementary file 2**: Variables for knee and shoulder cohorts. Contains detailed list of variables collected for each cohort.

## Data Availability

The data that support the findings of this study are stored in a secure research database that is not publicly available, and with restrictions around the availability of data so as not to compromise the privacy of personal and health information of patients.

## Abbreviations

PROM: Patient reported outcome measure
IQR: Interquartile range
BMI: Body mass index
ACORN: Arthroplasty Clinical Outcomes Registry New South Wales
