# Supplementary material for: Implementation and quality assessment of a clinical orthopaedic registry in a public hospital department
Source: BMC Health Serv Res. 2020 May 9;20:393. doi: 10.1186/s12913-020-05203-8 (PMC7210668; doi:10.1186/s12913-020-05203-8)
Supplement: Supplementary file 1 — Additional file 1: Supplementary file 1: Registry cohorts and inclusion/exclusion criteria. Defines cohorts for pilot registry and contains detailed list of inclusion and exclusion criteria for each cohort. [file 12913_2020_5203_MOESM1_ESM.pdf]

SUPPLEMENTARY INFORMATION

Supplementary file 1: Registry cohorts and inclusion/exclusion criteria

Table 1. Inclusion and exclusion criteria for registry cohorts

**Registry Cohort**	**Shoulder Instability**	**Rotator Cuff**	**Shoulder Arthritis**	**Proximal Humeral Fracture**	**Knee Instability**	**Knee Arthritis**
**Inclusion Criteria**	- Recent dislocation<br>- Traumatic capsuloligamentous injury<br>- Dislocation with glenoid fracture<br>- Atraumatic or voluntary dislocations	- Rotator cuff tear<br>- Subacromial impingement<br>- Cuff arthropathy	- Glenohumeral arthritis that has failed conservative management strategies	- Fracture requiring total shoulder arthroplasty	- ACL rupture that has failed conservative management strategies	- Failing conservative management for knee arthritis and opting to undergo total knee replacement
**Procedures**	- Stabilisation<br>- Bankart repair<br>- Latarjet<br>- Labral repair	- Rotator Cuff Repair<br>- Rev RCR<br>- Superior Capsular Reconstruction<br>- Rotator Cuff reconstruction<br>- Total Shoulder Arthroplasty (reverse)	- Total Shoulder Arthroplasty (anatomic)<br>- Total Shoulder Arthroplasty (reverse)	- Fracture fixation<br>- Total Shoulder Arthroplasty (reverse)	- ACL Reconstruction<br>- Rev-ACL Reconstruction	- Total Knee Arthroplasty<br>- Revision Total Knee Arthroplasty
**Exclusion Criteria**	- Associated rotator cuff tear<br>- Dislocation with associated proximal humeral fracture<br>- Instability with clinically important secondary arthritis	- Adhesive capsulitis<br>- Calcific tendonitis<br>- Advanced glenohumeral joint arthritis<br>- Tendinopathy<br>- Concomitant labral repair<br>- Inclusion in another cohort for a different index issue<br>- Dislocation with associated cuff tear<br>- Shoulder pathology associated with a dislocation event	- General exclusion criteria for the registry	- Concomitant glenoid fracture associated with an episode of dislocation<br>- Flake avulsions associated with rotator cuff tear or clavicle fracture	- Associated knee dislocation<br>- Requiring multiligament reconstruction or definitive treatment of two or more cruciate or collateral ligaments	- Patients treated with partial knee replacement
