# Supplementary material for: Implementation and quality assessment of a clinical orthopaedic registry in a public hospital department
Source: BMC Health Serv Res. 2020 May 9;20:393. doi: 10.1186/s12913-020-05203-8 (PMC7210668; doi:10.1186/s12913-020-05203-8)
Supplement: Supplementary file 2 — Additional file 2: Supplementary file 2: Variables for knee and shoulder cohorts. Contains detailed list of variables collected for each cohort. [file 12913_2020_5203_MOESM2_ESM.pdf]

SUPPLEMENTARY INFORMATION

Supplementary file 2: Variables for knee and shoulder cohorts

Table 1. Dataset variables common across all registry cohorts.

**Data linkage and demographics**	**Clinical**	**PROMS**	**Imaging**	**Complications**
First name	Date of initial examination	VR-12 - MCS	Image modality	Occurrence
Last name	Significant comorbidities	VR-12 - PCS	Date of study	Date of occurrence
ID	Consulting Surgeon		Provider	Nature
Date of birth	Final Diagnosis/Diagnoses		Report	Outcome
Age at initial exam	Surgery Type			
Gender	Treatment date			
Height	Hospital			
Weight	Assistant			
BMI	Anaesthetist			
Postcode				
Email				

Table 2. Cohort specific dataset variables.

Shoulder Instability	Rotator Cuff	Shoulder Arthritis	Proximal Humeral Fracture	Knee Instability	Knee Arthritis
SANE	SANE	SANE	VR-12	Tegner	VAS
Constant	Constant	Constant	Constant Score	IKDC	OKS
OSIS	WORC	WOOS	SANE	Passive Flexion	Passive Flexion
External Rotation	External Rotation	External Rotation	OSS	Fixed Flexion	Fixed Flexion
Internal Rotation	Internal Rotation	Internal Rotation	External Rotation	Lachman	Operating Surgeon
Strength of Abduction	Strength of Abduction	Strength of Abduction	Internal Rotation	Operating Surgeon	Tourniquet
Position	Position	Position	Strength of Abduction	Approach	Anaesthetic
Operating Surgeon	Operating Surgeon	Operating Surgeon	Position	Anaesthetic	Approach
Approach	Approach	Approach	Operating Surgeon	Tourniquet	Open
Open	Block type	Open	Approach	ACL	Alignment referencing
Block type	Anaesthetic	Block type	Block type	Condition	Bone Quality
Anaesthetic	Forward Elevation	Anaesthetic	Anaesthetic	Grade	Soft Tissue Release
Forward Elevation	ER at 90abd	Forward Elevation	Portals	Treatment	Patella Tracking
ER at 90abd	Cuff status	ER at 90abd	ASA rating	Graft side	Ligament Balance
Pathology found	Tendons Involved	Pathology	Bilateral (yes/no)	Graft type	Femur Brand
Location of tear	Tendon Quality	Cause of Osteoarthritis	Forward Elevation	Tendon type	Femur Fixation
Repair performed		Walch transverse Morphology	ER at 90abd	Fixation Femur	Femur Size
Suture Configuration		Rotator cuff tear	Pathology	Fixation Tibia	Tibia Brand
Anchor Type		Tendons torn	Post fracture injury cause	Graft size-Distal	Tibia Size

Labral Anchor Position		Procedure	Procedure	Proximal	Tibia Fixation
Procedure name		Retroversion	Retroversion	Notchplasty	Tibiofemoral Insert thickness
		Stability	Stability	Class	Patella button size
		Head Height in relation to Greater Tuberosity	Head Height in relation to Greater Tuberosity	Zone	Type
		ER	ER	Thickness	Patella Intervention
		Flexion	Flexion	Cause of tear	Constraint
		Brand	Brand	Location	Verasense Balancing
		Stem	Stem	Treatment	Tensiometer Balancing
		Body	Body	Meniscectomy	Manual Balancing
		Liner	Liner	Repair	Verasense report
		Component Type	Component Type	Class	NAV report
		Fixation	Fixation	Zone	Surgery/Procedure name
		Brand	Brand	Thickness	
		Size	Size	Cause of tear	
		Eccentricity	Eccentricity	Location	
		Type	Type	Treatment	
		Brand	Brand	Meniscectomy	
		Glenoid Size	Glenoid Size	Repair	

		Glenosphere	Glenosphere	Surgery/Procedure name	
		Liner	Liner		
		Fixation	Fixation		
		Glenoid Type	Glenoid Type		
		Method	Method		
		Type	Type		
		Procedure name	Procedure name		
